# Atlanta Society of Medicine

**Published:** 1897-07

**Authors:** 


### Society Reports.

### ATLANTA SOCIETY OF MEDICINE.

Atlanta, Ga., June 1, 1897.

Regular meeting of the Atlanta Society of Medicine.

In the absence of the President, Dr. Kendrick was asked to occupy the chair.

The following members were present: Drs. Bourns, J. E. Chappell, Hardon, Hutchins, Kendrick, Stirling, Stockard, VanDyke, Champion, Hancock.

The minutes of the previous meeting were read and approved.

The application for membership of Dr. M. G. Campbell was favorably reported upon by the committee, and upon ballot he was elected to membership.

Dr. Westmoreland was expected to read a paper before the Society but was detained out of the city and, therefore, could not be present.

### REPORT OF CASES.

Dr. Stockard: I have here a tumor or sac which I removed from the uterus of a patient. The case occurred last fall. The lady had missed one or two menstrual flows; after which she had a constant bloody flow for five or six weeks. One day I was sent for very quickly and was told that she had discharged quite a large clot of blood and upon examination I found this to be so. I then made a vaginal examination and I found just within the uterus a sac or tumor, and after cutting it out, I ruptured it and found it was simply a sac with nothing in it but the fluid. The inside was perfectly smooth, as was also the outer side, except where it was attached to the uterus.

Dr. Hardon: I do not know that I can say positively what the case was which Dr. Stockard has just reported, but cases like it sometimes occur. I saw one several years ago which was very similar in all details, an apparent miscarriage with simply a sac of fluid. I suppose it would come under the head of blighted ovum; where the development is expended upon the membrane of the ovum without developing of the fetus. We know that the ovum sometimes undergoes strange development; where there is no fetus at all but not really hydatid development; where there is no trace of the ovum but a perfect amniotic sac. I have seen a number of these attached by one stem. There is sometimes a fleshy development where the ovum expends its power simply upon this with no fetus. These are expelled usually in the early months of pregnancy. I do not know why they occur; they are simply a freak of nature. I have no doubt that this case of Dr. Stockard's would belong in that category and would be properly considered as blighted ovum. We know that in some cases where the fetus is formed there is a non-development of certain parts without apparent cause. The fetus may be born without head, limbs, and there may be other monstrosities of that sort, and this non-development may be so extensive as to involve not only certain parts but the entire fetus.

DR. HUTCHINS: I did not expect to report anything to-night and would simply mention a case which I saw this afternoon. A young woman about thirty years of age, who has a large tumor, about half as large as a child's head, growing just below the left clavicle and attached to the second, third and fourth ribs. She does not seem to know exactly how long it has been there, but it appears from the history which she gives that it began growing about ten years ago. It began as an immovable hard lump and grew slowly and without pain until about one year ago when it began to grow rapidly, and at present is about half the size of a child's head, and now there is sometimes pain in the tumor and surrounding tissue. Judging from its physical character I would say that it is an osteo-sarcoma. It will have to be operated upon if anything is done and a radical operation would be very formidable, as you all know.

DR. HARDON: I have had two cases recently which had a bearing upon the much-mooted and disputed question of performing operations for hystero-epilepsy. Some years ago it was considered that the operation was called for under those conditions, but experience showed that in many such cases which apparently demanded such operations, the expected benefit did not follow.

The two cases I have had recently occurred at the Grady Hospital. One was a woman thirty years of age who had had epileptic attacks for several years. They occurred first at the menstrual period and then sometimes between. It was true epilepsy with unconsciousness and frothing at the mouth. During one of the attacks she had fallen into the fire and her face was burned and one eye destroyed. She came to the hospital for relief and I mentioned to her that the removal of the ovaries might produce an improvement, as the attacks had been associated with the periods. I told her that there was no positive proof of relief but she decided to undergo the operation if there was any possible chance. I operated about two weeks ago and removed both ovaries, and found one to contain a cyst of considerable size, about as large as a walnut. The other ovary was normal. She recovered and since then has had no attack, except at the time when the last period should have come there was a slight momentary unconsciousness, but with that exception there has been no recurrence of the attacks.

The other case was a girl fifteen years of age who had menstruated for two years and had had epileptic attacks since the first period, after which the attacks became frequent. They have been confined to the periods to a great extent, although they sometimes came between them. When she came to the hospital they had become so frequent as to occur every day. I made an examination and found an infantile uterus. I explained to her and her friends the possibility of relief, and also the lack of certainty, but they were very desirous of the operation being performed. I operated three weeks ago and the night after the operation she had one epileptic seizure and since then she has had none at all. How long this relief will continue it is impossible to state. Experience shows that it is relieved for a time but may ultimately return. But when we consider the serious character of epilepsy, the liability to disease, and how it blights the patient's whole life, destroying the capacity for business and almost everything else, I think I was justified in operating with a mere possibility of relief, and thus far I am sure the results have justified the course I pursued. I know there are those who decry this operation, and I have seen cases where relief was not given in which I myself expected that it would be. I know that if I was one of these patients that I would catch at any straw; the patients felt that way and I performed the operations under those circumstances. However, I realize that it is too soon to know what will be the permanent results in these cases.

DR. KENDRICK: I remember Dr. Battey operated on all these cases. Did he carry this out to the end of his life?

DR. HARDON: Up to five years ago he operated upon all such cases. He was not much given to reporting his cases and I can not say whether he kept it up, but as long as I was familiar with his line of work I know that he did operate constantly on these cases.

DR. HANCOCK: Did you remove the normal ovary in the first case?

DR. HARDON: Yes, I believed that there would be greater certainty of relief by removing both, and also as the patient had requested it. In the case of the child fifteen years of age I attributed the condition to the undeveloped condition of the ovaries, and the uterus was infantile, not over an inch or an inch and a half in length.

Under the head of miscellaneous business, Dr. Hancock stated that, in accordance with the resolution at last meeting, his committee had placed the matter of prosecuting the unregistered doctors in the hands of Messrs. Andrews & Davis, and that these gentlemen had agreed to get up cases for the fees which they expected to get out of it, but they had to pay witnesses to get up evidence and requested the Society to advance $10.00 for this purpose, and if they collected any fees this amount would be refunded to the Society.

This was laid over until next meeting.

It was mentioned that the adjournment of the Society for the summer would be at the next meeting and, therefore, Dr. Hancock made a motion that, as the Society was renting its present quarters by the month, they give up same after next meeting and store the chairs, etc., until the first of September. Motion carried.

